# Body Size Evolution in Insular Speckled Rattlesnakes (Viperidae: *Crotalus mitchellii*)

**DOI:** 10.1371/journal.pone.0009524

**Published:** 2010-03-04

**Authors:** Jesse M. Meik, A. Michelle Lawing, André Pires-daSilva

**Affiliations:** 1 Department of Biology, The University of Texas at Arlington, Arlington, Texas, United States of America; 2 Department of Geological Sciences, Indiana University, Bloomington, Indiana, United States of America; Université de Montpellier 2, France

## Abstract

**Background:**

Speckled rattlesnakes (*Crotalus mitchellii*) inhabit multiple islands off the coast of Baja California, Mexico. Two of the 14 known insular populations have been recognized as subspecies based primarily on body size divergence from putative mainland ancestral populations; however, a survey of body size variation from other islands occupied by these snakes has not been previously reported. We examined body size variation between island and mainland speckled rattlesnakes, and the relationship between body size and various island physical variables among 12 island populations. We also examined relative head size among giant, dwarfed, and mainland speckled rattlesnakes to determine whether allometric differences conformed to predictions of gape size (and indirectly body size) evolving in response to shifts in prey size.

**Methodology/Principal Findings:**

Insular speckled rattlesnakes show considerable variation in body size when compared to mainland source subspecies. In addition to previously known instances of gigantism on Ángel de la Guarda and dwarfism on El Muerto, various degrees of body size decrease have occurred frequently in this taxon, with dwarfed rattlesnakes occurring mostly on small, recently isolated, land-bridge islands. Regression models using the Akaike information criterion (AIC) showed that mean SVL of insular populations was most strongly correlated with island area, suggesting the influence of selection for different body size optima for islands of different size. Allometric differences in head size of giant and dwarf rattlesnakes revealed patterns consistent with shifts to larger and smaller prey, respectively.

**Conclusions/Significance:**

Our data provide the first example of a clear relationship between body size and island area in a squamate reptile species; among vertebrates this pattern has been previously documented in few insular mammals. This finding suggests that selection for body size is influenced by changes in community dynamics that are related to graded differences in area over what are otherwise similar bioclimatic conditions. We hypothesize that in this system shifts to larger prey, episodic saturation and depression of primary prey density, and predator release may have led to insular gigantism, and that shifts to smaller prey and increased reproductive efficiency in the presence of intense intraspecific competition may have led to insular dwarfism.

## Introduction

The striking phenotypic divergence often exhibited by island populations when compared to their putative mainland ancestors has long attracted the attention of evolutionary biologists [1], [2]. Factors affecting phenotypic evolution on islands have been attributed to both founder effects and exposure to fundamentally different selection regimes, which often include ecological release from competition and predation as well as severe resource limitation [3], [4], [5]. Furthermore, many factors are not mutually exclusive and evolutionary changes initially resulting from genetic drift within small founding populations may also provide the impetus for selection to drive phenotypic evolution towards a different adaptive peak [6]. The combination of both adaptive and nonadaptive forces acting concurrently may produce especially rapid rates of phenotypic evolution when compared to mainland source populations [7], [8].

Although many extreme modifications to behavior and morphology among island populations seem unique and system-specific, large-scale patterns are also evident and attest to common underlying evolutionary processes. One of the large-scale patterns of phenotypic variation in insular vertebrates, known as the island rule [4], [9], describes the tendency for small-bodied island founders to increase in size (insular gigantism) and for large-bodied island founders to decrease in size (insular dwarfism). This pattern has been observed across many vertebrate taxa (see [4]) and has been used in support of evolutionary concepts such as optimal body size [10], [11]. However, many recent studies based on large datasets have found little support for the island rule for many vertebrate groups (e.g., [12], [13]). It seems from the disparity of results among such studies that factors affecting body size evolution in island populations are complex. Apparent discrepancies in the observed patterns may partly reflect clade-specific responses to underlying ecological factors, adding an additional level of complexity to interpretations of insular body size shifts [14].

Intraspecific comparisons between mainland source species and their derivative populations on multiple islands have also revealed complex patterns of body size variation (e.g., [12], [15], [16], [17]). Many of these studies examined the relationship between body size and three island physical characteristics: area, age, and distance from mainland source. Because these variables only indirectly influence body size evolution, the underlying factors remain speculative. If drift or non-uniform selection pressures dominate across islands, then no relationship with body size is predicted. If different body size optima are related to suites of selective pressures that differ as a function of island area, then body size is predicted to correlate with island area [12], [16], [17]. Island area effects should be more pronounced on smaller islands, as larger islands more closely approximate continental conditions in terms of species richness and resultant community dynamics. Distance to mainland may influence body size because presumed higher immigration rates to near-shore islands would dilute *in situ* divergence through influx of gene flow from the mainland [3]. Finally, island age may show a relationship with body size when consistent directional selection for either dwarfism or gigantism is still acting in island populations. For example, Anderson and Handley [17] demonstrated a negative relationship between island age and body size in three-toed sloths (*Bradypus* spp.) from the Bocas del Toro Islands of Panama, suggesting evolutionary disequilibrium between directional selection for dwarfism and the temporal scale required to obtain optimal body size following isolation of land-bridge islands since the last glacial maximum.

Several studies have documented replicated instances of insular gigantism and dwarfism (or both) among insular populations of squamate reptiles (e.g., [11], [18], [19], [20], [21]). In studies of lizard body size distributions, Meiri [13], [22] did not detect a clear bias for insular gigantism over dwarfism among insular lizards, but found that island populations tend to occupy extreme ends of the global lizard body size distribution. Body size shifts are also common in island snake populations, and have been explained by changes in available prey, decreased intensity of interspecific competition, and relaxed predation pressures [23], [24]. Across snake species there is a tendency for populations that are dwarfed on islands to specialize on small lizard prey and for snakes that are giant to occupy islands that support colonies of nesting seabirds, which provide a seasonally available food source [23], [25]; however, there are exceptions to this pattern (e.g., [26]). Collectively, these findings suggest that while selective pressures on islands clearly influence body size evolution, ultimate mechanisms are likely to defy simple explanations.

**Figure 1:**
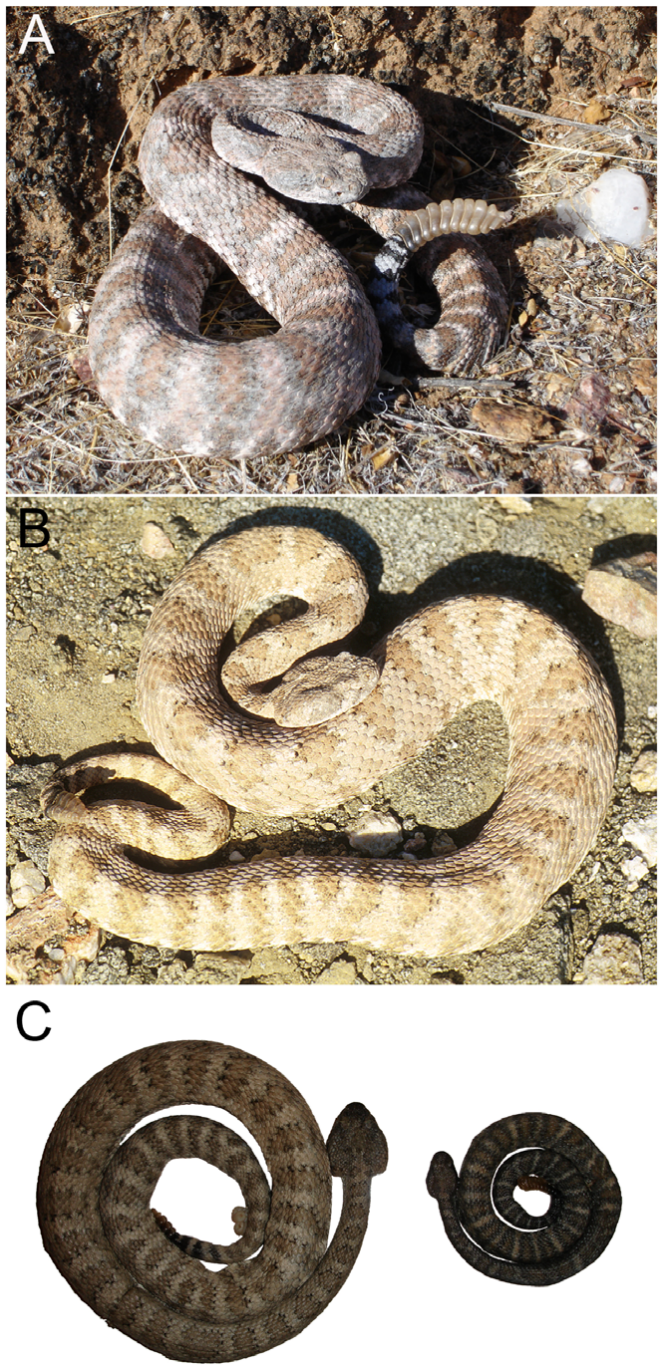
Photos in life of a typical adult speckled rattlesnake from Isla El Muerto (A) and a typical adult speckled rattlesnake from Isla Ángel de la Guarda (B) (both males). (C) Preserved specimens from A (right) and B (left) photographed to scale, showing size difference.

**Figure 2:**
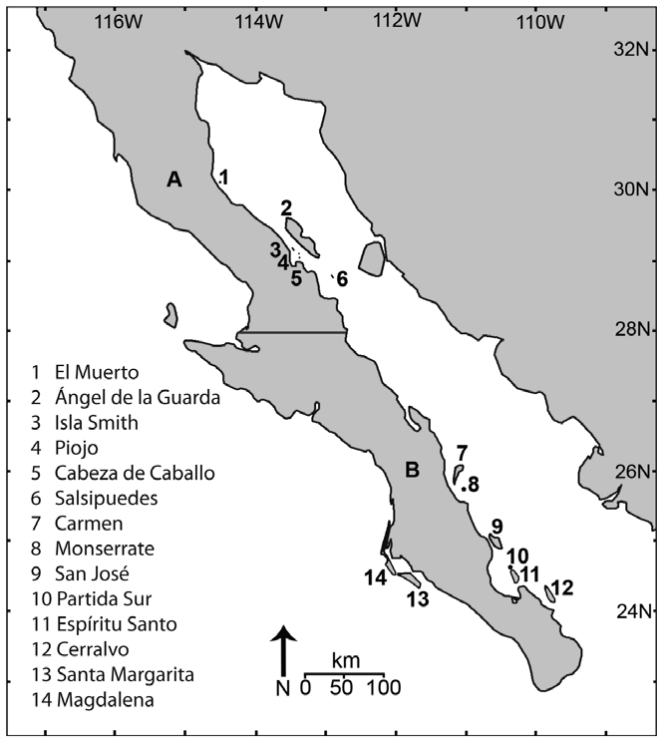
Map of the Baja California peninsular region depicting locations of islands inhabited by speckled rattlesnakes in the Sea of Cortés and Pacific Ocean. The solid line bisecting the peninsula indicates the political boundary between Baja California and Baja California Sur and the approximate boundary between the mainland subspecies *Crotalus mitchellii pyrrhus* to the north (A) and *C. m. mitchellii* to the south (B).

Among viperid snakes, and rattlesnakes in particular, insular dwarfism tends to be the rule [23], [24]. The only reported instance of insular gigantism in a viper is from the population of speckled rattlesnakes (*Crotalus mitchellii*) on Isla Ángel de la Guarda in the Sea of Cortés [24], [27] (Fig. 1). Dwarfed speckled rattlesnakes inhabit Isla El Muerto, also in the Sea of Cortés [28] (Fig. 1). *Crotalus mitchellii* has been reported from 12 additional islands off the coast of peninsular Baja California [29], [30] (Fig. 2); however, no comprehensive study of body size variation exists. Here we document body size variation among island populations derived from mainland *C. mitchellii* and relate these data to hypotheses based on predictions from island physical variables. We also compare allometric differences in relative head size between samples of dwarfed (El Muerto) and giant (Ángel de la Guarda) speckled rattlesnakes, and their putative mainland source clade (*C. m. pyrrhus*). Because snakes are gape-limited predators, differences in the allometry of relative head size may provide evidence consistent with predictions based on shifts in prey (diet alteration) as a selective factor for body size change [31], [32]. Increasing evidence supports gape size, rather than body size, as a more direct target for selection tracking diet shifts in insular snakes [26], [33].

## Materials and Methods

### Island Data

The peninsula of Baja California extends for approximately 1250 km in a northwest-southeast trajectory, separated from the western coast of mainland Mexico by the Sea of Cortés. Most of the peninsula is arid and shares faunal and floral affinities with both the Sonoran Desert of northwestern Mexico and the southwestern United States, and subtropical thornscrub of western Mexico [29]. As the site of extensive volcanism and tectonic events, the geological history of the region has been exceptionally dynamic over the past 10 my. Rifting of what is now the San Andreas Fault system eventually separated the peninsula from mainland Mexico approximately 4–6 mya as the Pacific Plate migrated in a northwesterly direction [34], [35], [36]. Islands occur along the length of both sides of the peninsula, but are especially numerous in the western Sea of Cortés. Although a few islands are likely oceanic in origin, most were either sheared from the peninsula by tectonic activity as the peninsula moved in a northwesterly direction, or are land-bridge islands—peninsular fragments that have been isolated for no longer than 15 ky [36]. In general, islands are similar in flora, climate, and topography [37].

We collated data for each of the following three physical characteristics for islands occupied by *C. mitchellii*: island area, island distance, and island age (Table 1). Both island distance and island age are considered measures of isolation. Here, island distance refers to geographical isolation and was measured as the straight-line distance (in km) of an island from the Baja California peninsula. Island age refers to temporal isolation and was measured as the estimated time lapse (in years) since an island shared a physical connection with the peninsula. Estimates of island area and distance were obtained from Murphy et al. [38]. Estimates of island age were obtained from Carreño and Helenes [36], Wilcox [39], and using the eustatic sea level curve of Milliman and Emery [40], based on minimum channel depths between islands and the peninsula.

**Table 1:** Means of SVL and sample sizes (*N*) for island and mainland speckled rattlesnakes and descriptive data for inhabited islands.

Island Population/Mainland Subspecies	*N*	Mean SVL (mm)	Island Age (ky)	Distance to Mainland (km)	Area (km^2^)
El Muerto*	34	516.5	8.3	3.39	1.33
Angel de la Guarda*	42	947.3	1500	12.12	930.07
Smith*	6	621.2	7.7	2.18	8.91
El Piojo*	5	517.3	8.3	2.61	0.55
Salsipuedes	1	775.0	1500	16.36	1.08
Carmen	3	723.5	15	6.03	140.84
Monserrate	6	711.7	4000	13.7	19.86
San Jose*	8	698.0	10.6	4.16	174.71
Espiritu Santo*	8	684.8	6.9	6.15	84.08
Partida Sur*	5	582.2	7.0	6.15	19.29
Cerralvo	4	766.3	2000	8.73	140.17
Margarita	1	776.0	4.0	7	231
*C. m. pyrrhus*	246	794.0	-	-	-
*C. m. mitchellii*	68	786.4	-	-	-

Asterisks indicate island populations that are significantly different from mainland rattlesnakes in SVL (random effects ANOVAs).

### Morphological Data

We acquired morphological data from whole ethanol-preserved specimens of *C. mitchellii* housed at various natural history collections in the United States and Mexico (Appendix S1; *N* = 437 specimens). Specimens originated from throughout the mainland distribution and from 12 of the 14 inhabited islands. Museum abbreviations follow Leviton et al. [41], except as indicated in Appendix S1. Adult and near-adult specimens were selected on the basis of locality data availability and whether condition of specimens was suitable for accurately recording morphological variables. Ontogeny was roughly estimated by examining the rattle structure for near parallelism of successive rattle segments indicating that growth rates were asymptotic at the time of preservation [42]. Sex was determined by evaluating presence of hemipenes. For each specimen we measured snout−vent length (SVL) to the nearest 1 mm using a string and metric rule and obtained a separate head length measurement to the nearest 0.1 mm using a digital caliper. Body length was obtained by subtracting head length from SVL.

### Analyses

Two subspecies of *C. mitchellii* are widely distributed throughout peninsular Baja California: *C. m. pyrrhus* occupies the northern half of the peninsula and *C. m. mitchellii* occurs throughout the southern half. These subspecies show correspondingly deep phylogenetic structure based on mitochondrial DNA sequences and represent distinct clades [43]. For all analyses we assumed that island populations were most closely related to the nearest mainland subspecies of *C. mitchellii*. This assumption is concordant with preliminary molecular data (JMM, unpublished data). We considered all island populations to be *C. mitchellii* regardless of the possibility of peripatric speciation. Owing to the difficulty in obtaining specimens, sample sizes of snakes from island populations were relatively low (mean *N* = 10.3; mean *N* = 4.7 when El Muerto and Angel de la Guarda are excluded). Minor male-biased sexual size dimorphism occurs in mainland *C. mitchellii*. Variation in bias and magnitude of sexual dimorphism may occur in island populations (e.g., [44], [45], [46]); however, our sample sizes across islands were not sufficient to adequately address this phenomenon. Because sex ratios were similar across most populations, we combined data from males and females to increase statistical sampling unless otherwise noted. The possible influence of sexual dimorphism would be amplified on islands with very low sample sizes; therefore, we included in statistical analyses only island populations from which we had obtained at least three adult individuals. Omitting islands with low sample sizes also reduced the potential influence of specimens that may have erroneous locality data. All statistical analyses were conducted using Systat 12.

We performed random-effects ANOVAs to evaluate differences in mean SVL of speckled rattlesnakes between each mainland source subspecies and its respective island populations. We used mean SVL to reduce biases that result from large discrepancies in sample sizes [13], and adjusted alpha using Bonferroni correction for multiple comparisons. A multiple regression approach using Akaike's information criterion (AIC) model selection was employed to compare a set of 9 *a priori* candidate models using log_10_-transformed values for the three island physical characteristics as independent variables and log_10_ mean SVL as the dependent variable. Island populations are derived from two distinct mainland clades, therefore phylogenetic nonindependence could influence regressions. In this instance we considered the potential influence to be negligible because the two mainland subspecies differed in SVL by less than 8 mm (Table 1); thus, we included all island populations in the regression models. Candidate models incorporated all combinations of main effects, additive effects, and interactions up to five total parameters. We used a small sample size correction (AIC_c_) as recommended by Burnham and Anderson [47]. We ranked relative support for the various regressions by comparing ΔAIC_c_ of the best approximating model (AIC_min_) and each competing model (AIC*_i_*). Values between 0−2 for ΔAIC_c_ indicate similar support [47]. We further evaluated model fit using weights (*W_AIC_*), which are the relative likelihoods of each model given the data.

We compared scaling relationships of relative head length among rattlesnakes from Ángel de la Guarda (insular giants), El Muerto (insular dwarfs), and their putative mainland source subspecies (*C. m. pyrrhus*) using ANCOVAs. We did not include data from other island populations because of small sample sizes. For this analysis, we included log_10_ head length as the dependent variable and log_10_ body length (SVL – head length) as the covariate. We first tested for homogeneity of slopes; in the event of non-significance we further tested for a difference in intercepts, with *F*-tests based on type III sums of squares. To reduce the influence of sexual dimorphism we analyzed males and females separately. Because of the exploratory purpose of this analysis, we set alpha  = 0.10 for all comparisons to better detect biologically relevant patterns in head size [48].

## Results

**Figure 3:**
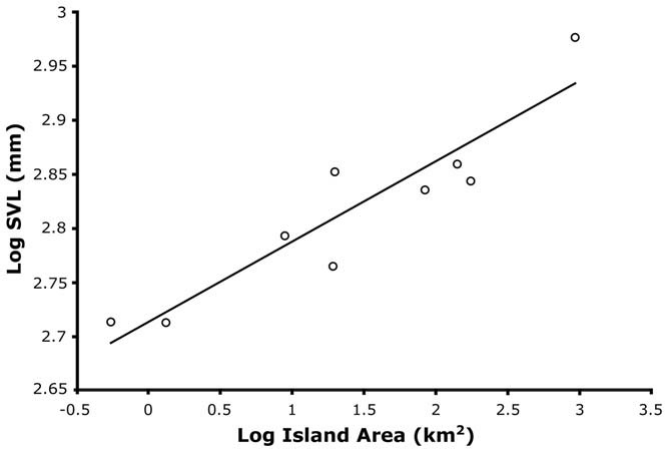
Scatterplot of log SVL means for island populations of speckled rattlesnakes as a function of log island area. The *R^2^* value for this model (including only area as a predictor variable) equals 0.84.

Mean SVL of island samples of *C. mitchellii* ranged from 516.5−947.3 mm (Table 1). With the exception of insular gigantism on Ángel de la Guarda, all island populations showed a tendency towards dwarfism; six of which were significantly lower in mean SVL than their putative mainland source subspecies (Table 1). The smallest rattlesnakes occurred on El Muerto, Piojo, and Partida Sur, which are all land-bridge islands sharing a recent connection to the peninsular mainland. The strongest competing model for the influence of island physical variables on mean SVL included area and island age (Table 2). The sum of the *W_AIC_* for models which included area equaled 1.0, providing strong support for the relative importance of area as a predictor of SVL. The model including only island area had *R^2^* = 0.84 (Fig. 3). Models including island age, but excluding island area, had a sum of *W_AIC_* values equal to 0, indicating that the inclusion of island age in the best competing model was likely a spurious effect of the inverse correlation between island area and island age (i.e., most small islands are also land-bridge islands, and are therefore young). Distance to mainland as a predictor of SVL was not strongly supported by any model.

**Table 2:** Model selection results for nine candidate models using mean SVL of island populations with sample sizes ≥3 as the response variable and three island physical characteristics as predictor variables.

Model	*K*	*R^2^_Adj_*	ΔAIC_C_	*W_AIC_*
Area + Age	4	0.94	0	0.78
Area	3	0.84	3.81	0.12
Area + Age +Area*Age	5	0.94	4.95	0.07
Area + Distance	4	0.86	6.93	0.02
Area + Distance + Area*Distance	5	0.91	9.00	0.01
Distance	3	0.49	15.59	0.00
Age	3	0.40	17.35	0.00
Distance + Age	4	0.44	21.20	0.00
Distance + Age + Distance*Age	5	0.39	29.37	0.00

Models are ranked by ΔAIC_C_. *K* =  the total number of parameters in each model; *W_AIC_* is the Akaike weight. Area  =  island area, Age  =  island age, Distance  =  distance to peninsular mainland.

With respect to ANCOVA results comparing relative head length, neither males nor females from Ángel de la Guarda showed interaction effects with mainland *C. m. pyrrhus* for the test of homogeneity of slopes (Table 3). Snakes from Ángel de la Guarda had relatively larger heads than did mainland *C. m. pyrrhus*, though this pattern was only marginally significant for males. A significant interaction effect was detected between males from El Muerto and males from the mainland (Table 3), with males from El Muerto having a lower slope. The null hypothesis of homogeneity of slopes was not rejected for females from El Muerto and the subsequent intercept test indicated that females from El Muerto had relatively smaller heads than mainland *C. m. pyrrhus* (though only marginally significant).

**Table 3:** Means and coefficients of variation for raw head length measurements for giant (Ángel de la Guarda), dwarfed (El Muerto) and mainland (*C. m. pyrrhus*) speckled rattlesnakes.

						Slope			Intercept		
		HL	CV	MHL	CV	df	*F*	*P*	df	*F*	*P*
Ángel de la Guarda											
	Males	44.5	0.23	35.9	0.17	1	0.036	0.849	1	3.136	0.079
	Females	36.5	0.27	31.0	0.15	1	1.636	0.204	1	6.297	0.014
El Muerto											
	Males	25.7	0.09	35.9	0.17	1	12.025	0.001	-	-	-
	Females	23.6	0.09	31.0	0.15	1	1.366	0.246	1	2.958	0.089

Also provided are ANCOVA results for tests of homogeneity of slopes and difference in slopes.

HL  =  head length, MHL  =  mainland head length (*C. m. pyrrhus*). Sample sizes: 141 for *C. m. pyrrhus* males, 20 for *C. m. angelensis* males, 15 for *C. m. muertensis* males, 105 for *C. m. pyrrhus* females, 22 for *C. m. angelensis* females, and 19 for *C. m. muertensis* females.

## Discussion

### Body Size Variation

Insular speckled rattlesnakes tend towards decreased body size when compared to mainland conspecifics. Because dwarfed *C. mitchellii* occurs on small, recently isolated, near-shore islands, multicollinearity of island physical variables that may influence SVL is problematic. Also, because we did not evaluate sexual size dimorphism, differences in sex ratios of samples could affect regression results; therefore, we interpret results cautiously. AIC model selection considered in the context of hypothetical predictions supports island area as the most important predictor of body size (Table 2; Fig. 3). Distance to mainland was not supported by any model, and island age was supported only by a model that included island area. Furthermore, the most divergent rattlesnake samples originated from land-bridge islands, a pattern that is opposite to *a priori* predictions of the influence of island age on body size evolution (i.e., more recently isolated islands should show less divergence). Although relationships between body size and island area have been discussed repeatedly over the last few decades, they have received relatively little empirical support (e.g., [12], [17], [49]). Relationships between body size and island area have been reported for relatively few mammal species [16], [50], [51]. Among squamate reptiles, island area effects on intraspecific size variation have been studied only in side-blotched lizards (genus *Uta*), which do not seem to co-vary in body size as a function of island area [15], [45]. In a large-scale study that included *C. mitchellii*, Boback [24] concluded that island area was not supported as a determinant of body size shifts in island snakes; however, it is likely that the scale of his analysis precluded an adequate evaluation of intraspecific patterns.

Collective evidence suggests that selection, rather than drift, is the main evolutionary force underlying patterns of body size variation among insular speckled rattlesnakes. A nonrandom relationship between body size and island area, as seen here, is an explicit prediction of selection for optimal body size due to indirect island area effects on resource availability and community composition [4], [16], [17]. The influence of island area should be strongest on smaller islands and accordingly speckled rattlesnakes show a trend towards dwarfism on islands that are smaller than about 20 square kilometers (Table 1). Many studies have inferred selection (and ruled against drift) using signed rank tests (e.g., [17], [52]), but these tests are inconclusive in that failure to reject the null hypothesis could indicate the influence of either genetic drift or opposing selection pressures across different islands. Furthermore, drift may lead to directional change if mutations affecting body size are biased towards a particular direction (although one would not necessarily predict a strong relationship between body size and island size based on such a bias).

The smallest speckled rattlesnakes occur on El Muerto and El Piojo. Both islands separated from the mainland approximately 8.3 kya, indicating that body size shifts can occur rapidly. Based on dwarfed rattlesnake occurrence on land-bridge islands and the considerable distance between most islands harboring dwarfs, it is likely that these shifts in body size represent independent evolutionary events. The single known island harboring giant rattlesnakes, Ángel de la Guarda, was severed from the peninsular mainland by rifting of the San Andreas Fault system approximately 1.5 mya and is surrounded by deep water; thus, this population likely has been isolated from mainland ancestors nearly 200 times longer than the land-bridge island populations of dwarfed rattlesnakes. The two oldest islands that are inhabited by *C. mitchellii* (Islas Cerralvo and Monserrate) do not have rattlesnakes that deviate significantly in SVL from the mainland. A seemingly parallel pattern of rapid body size evolution including both dwarfism and gigantism has been reported for tiger snakes (genus *Notechis*) from islands off the coast of southern Australia [20].

Measures of length are standard proxies of body size in snakes but do not adequately reflect the magnitude of body size differences between the smallest and largest speckled rattlesnakes. Rattlesnakes from El Muerto and El Piojo are diminutive in both length and girth; one female (CAS 146566, El Piojo) of only 360 mm SVL had developing follicles, indicative of sexual maturity. A sample of three adult male rattlesnakes from El Muerto averaged 70.2 grams in mass (JMM unpublished data). In contrast, individuals from Ángel de la Guarda may exceed 1200 mm in length and 1.5 kg in mass [27][JMM unpublished data]. Although it is clear that there must be differences in selective forces influencing dwarfism and gigantism, the ultimate mechanisms underlying such dramatic divergence in body size remain speculative.

### Possible Selective Forces

Several studies have sought to explain body size evolution in insular snake populations (e.g., [23], [24], [53], [54]); however, in most cases assessments of selective factors have involved only coarse data (e.g., lists of potential competitor and predator species). Most of these studies have implicated diet alteration, as opposed to competition or predator release, as the primary factor influencing body size evolution (but see [23]). We consider this conclusion to be premature as most researchers have not performed detailed dietary comparisons nor have they adequately ruled out competition or life history shifts resulting indirectly from predator release. Many studies dismissing competition (e.g., [24], [46]) have implicitly considered only interspecific competition as opposed to intraspecific interactions, or perhaps more importantly, the relative strength of interspecific versus intraspecific competition.

The ANCOVA results comparing relative head size between *C. m. pyrrhus* and rattlesnakes from El Muerto and Ángel de la Guarda revealed that rattlesnakes from El Muerto had comparatively lower growth rates in relative head length (at least among males), consistent with selection for smaller gape size associated with shifts to smaller prey. Case [23] noted that rattlesnakes from the Sea of Cortés tended to dwarf on islands where the relative abundance of small lizards was greater than rodents, implying that the overall size distribution of prey had shifted towards a smaller mode. We did not reject the null hypothesis of no difference in slopes between head length of mainland *C. m. pyrrhus* and rattlesnakes from Ángel de la Guarda; however, when corrected for body length, the island snakes had larger heads. Although little is known of the natural history of Ángel de la Guarda speckled rattlesnakes, it is likely that they feed mostly on the giant endemic chuchwalla lizard, *Sauromalus hispidus* [23], which attains weights of up to 1.4 kg [53]. Case [23], [53] speculated that gigantism in speckled rattlesnakes was a compensatory response to increased mass of its primary prey. Our ANCOVA results indicating proportionately larger head size in the Ángel de la Guarda population support this hypothesis but with the viewpoint that increased gape size may have led to correspondingly large body size. *Sauromalus hispidus* also occurs on El Piojo and Smith Islands, which have dwarfed speckled rattlesnakes; however, the presence of this giant lizard on these islands may be a result of recent Seri Indian introductions (see [55]).

In an unusual case of body size reversal, dwarfed red diamond rattlesnakes (*C. ruber*) occur sympatrically with giant speckled rattlesnakes on Ángel de la Guarda (on the peninsula, *C. ruber* attains larger body size). Case [53] speculated that *C. mitchellii* colonized Ángel de la Guarda first and was able to exploit the giant chuckwalla as prey. When *C. ruber* later became established on the island it decreased in size by switching to a diet that would reduce interspecific competition. We offer an alternative hypothesis based on the more plausible scenario that ancestral populations of insular *C. mitchellii*, *C. ruber*, and *S. hispidus* were simultaneously isolated from the peninsular mainland by the separation of Ángel de la Guarda (e.g., vicariance as opposed to colonization). Compared to most other rattlesnakes (including *C. ruber*), *C. mitchellii pyrrhus* has a proportionately large head in both length and width dimensions [56], a morphological feature that may have predisposed this taxon, instead of *C. ruber*, to track the *in situ* evolution of increasing body size in chuckwallas. In response, *C. ruber* may have specialized on smaller prey, which would decrease interspecific competition as suggested by Case [53].

In addition to prey size, the temporal availability of prey may favor selection for gigantism in insular snakes. Because mass-specific metabolic rate decreases with increase in absolute mass, larger snakes are capable of greater fasting endurance, which would allow for increased survivorship on islands with both (or either) frequent fluctuations in densities and relatively high extinction rates of prey species. In a study of adders (*Vipera berus*) on islands in the Baltic Sea, Forsman [26] posited that populations obtained larger body sizes on islands with two potential prey species than on islands with three because greater starvation risks would be associated with fewer prey species. Giant tiger snakes (*Notechis*) from Chappell Island, Australia, consume mutton-bird chicks, which are a large, seasonally available, and saturating resource [25]. For tiger snakes, larger size not only confers fasting endurance but also presumably is associated with larger gape size, which would allow snakes to use the time-limited resource for longer periods before chicks fledge. A similar situation of alternating saturation and extreme limitation of food resources may prevail for giant speckled rattlesnakes. Data from a long-term study of *S. hispidus* population dynamics on Ángel de la Guarda revealed that densities fluctuate greatly from year to year, especially in response to El Niño climatic events [53]; thus, giant speckled rattlesnakes provide an additional example implicating fasting endurance as a potential selective force for larger body size in insular snakes.

Although shifts in diet and prey availability likely have influenced extant patterns of body size in speckled rattlesnakes, diet alteration alone is not sufficient to explain the clear relationship between log SVL and log island area. Island area may indirectly influence many aspects of community composition and resource dynamics in addition to prey size [16], [57]. Palkovacs [58] argued that life history shifts resulting from reduced extrinsic mortality (predator release) and resource limitation can favor either gigantism or dwarfism depending on the relative importance of these factors. In general, predator release is expected to result in increased body size while reduced resource availability is expected to result in decreased body size [58]. We presume extrinsic mortality rates to be low for rattlesnakes on Ángel de la Guarda, an island that has no native mammalian mesopredators [59]. Speckled rattlesnakes from Ángel de la Guarda have proportionately small rattles [27], which may reflect initial stages of vestigilization of the rattling system in the absence of predators. Furthermore, speckled rattlesnakes from Ángel de la Guarda are generally placid and reticent to rattle when disturbed, suggesting a relaxed antipredator behavioral response. Also, evidence from another giant snake population (*Elaphe quadrivirgata* on Tadanae-jima, Japan) suggests that large size is achieved gradually throughout ontogeny rather than by more rapid growth in juveniles, suggesting increased longevity (i.e., reduced mortality) in insular giants [60].

Intense intraspecific competition and high densities of conspecifics are general features of especially small, species-poor islands [4], [23]. Following Palkovacs [58], we contend that the relative importance of resource limitation over predator release in life history trait evolution increases on these ecologically simplified islands [23], [50], [57]. Density overcompensation has been documented for phyrnosomatine lizards on several small islands in the Sea of Cortés [15], [16]. Evidence from various insular snake populations (e.g., [61], [62]), including anecdotal evidence for *C. mitchellii* on El Muerto [28], suggests that density overcompensation may prevail on small islands for snake populations as well. Although we cannot directly address competition, *C. mitchellii* is smaller on islands where congeners are absent than when they are present (Mann-Whitney U-test, *P* = 0.021), suggesting that dwarfism occurs with greater frequency under conditions where strength of intraspecific competition is likely greater than interspecific competition.

Under intense intraspecific competition, populations may respond in three non-mutually exclusive ways. First, individuals may shift their diets to broaden exploitable niche space, which would result in increased intra-population variation in body size [44], [63]. This option is likely not available on small, species-poor islands. Second, individuals may employ a monopoly strategy and consume a greater proportion of the available resources. Adoption of this strategy would favor increased body size and fecundity, and has been suggested for some insular populations of birds, lizards, and mammals [64], [65], [66]. One caveat of the monopoly strategy is that it is predicted mostly for populations that experience interference, rather than exploitative, competition [23], [50], which is not typical of snakes. A third strategy would increase reproductive efficiency by diverting resources from somatic growth to reproductive output [4], [58]. This life history shift would result in decreased body size at reproductive maturity. Given that dwarfism occurs on small islands, and assuming that intraspecific competition is particularly intense under small island conditions, the third strategy is the only one of these three alternative hypotheses that is currently supported, and may have contributed to insular dwarfism in *C. mitchellii*.

Body size is fundamentally important to most aspects of life history and selective agents of body size variation are likely numerous [67]. We posit that shifts to larger prey, periodicity of prey densities, and predator release may have resulted in gigantism for speckled rattlesnakes on Ángel de la Guarda. In contrast, shifts to smaller prey and increased reproductive efficiency accompanied by strong intraspecific competition may have led to dwarfism among speckled rattlesnakes occupying small land-bridge islands. More direct measures of inter- and intraspecific competition and detailed investigations of dietary differences among island and mainland populations may provide further support for these hypotheses.

## Supporting Information

Appendix S1 Specimens examined and institutional abbreviations not included in Leviton et al. [41].
(0.04 MB DOC)
