# Supplementary material for: Body Size Evolution in Insular Speckled Rattlesnakes (Viperidae: Crotalus mitchellii)
Source: PLoS One. 2010 Mar 4;5(3):e9524. doi: 10.1371/journal.pone.0009524 (PMC2832004; doi:10.1371/journal.pone.0009524)
Supplement: Appendix S1 — Specimens examined and institutional abbreviations not included in Leviton et al. [41]. (0.04 MB DOC) [file pone.0009524.s001.doc]

Appendix S1

Total specimens examined (*N* = 571) from natural history collections. Abbreviations follow Leviton et al. (1985) except for the following: MBM = Marjorie Barrick Museum of Natural History, University of Nevada Las Vegas; UABC = Universidad Autónoma de Baja California; MZFC = Museo de Zoología, Facultad de Ciencias, Universidad Nacional Autónoma de México; IBH = Instituto de Biología Collecion de Herpetología.

*Crotalus mitchellii angelensis* (41): BYU 41133; CAS 50869–70, 50904, 103471, 142178; IBH 6152, 6299; MVZ 96803, 96805; MZFC 5012, 5015, SDSNH 19717–18, 19992–95, 44358, 48028–34, 51991–96, 53053; USNM 8562, 15978, 240906, 240908–09. MZFC uncatalogued (3 specimens).

*Crotalus mitchellii mitchellii* (96): BYU 34636–37, 34639–40, 34960, 41939; CAS 93, 14030–31, 45887, 52547, 52755, 52816, 52842, 52890, 53711, 101363, 102916, 103470, 146610, 192749; FRM 109, 209, 221, 311, 358, 459, 609, 688; IBH 1045, 1074, 2565, 2605, 3375, 3425; KU 173097; LACM 25083–85, 74029, 104962, 134439; MVZ 11923–24, 13793, 50177, 161442–43, 182176, 190033; MZFC 7597, 16663; SDSNH 2220–22, 2692, 6602–04, 17650–51, 20114, 20498–99, 20563–65, 20780–81, 20784, 20824, 20942, 20946, 21046, 21507, 21596, 22704, 22706, 23096–97, 44360–62, 44675, 45002, 52909, 61226, 68740; UABC 1101, 457; UCM 26136, 26273; USNM 12625, 240364–65, 240691.

*Crotalus mitchellii muertensis* (31): BYU 46318, 46341, 81355–63; KU 155535, 174830; SDSNH 37442–49, 38040, 48027, 56950, 56951; UABC 1494–95; UTA 7622. MZFC uncatalogued (3 specimens).

*Crotalus mitchellii pyrrhus* (269): ASU 1606, 2678, 3255, 4585, 9073, 15834, 15836–37, 23276, 24342, 34599, 34607, 34775; BYU 34507–10, 34613, 34667, 34702–03, 35960–61; CAS 10108, 17540, 19981–82, 20814, 40091, 90181, 90187, 103466, 143168, 143559, 146566, 146586, 182495, 182499, 182562, 191134, 192750–51, 192750–51, 192754–55, 201174, 214186; IBH 4208; KU 5335, 6996, 31356, 61314, 105920–21; LACM 19998, 20006, 28018, 52593–94, 59178, 75838, 104873–74, 104937–38, 104949, 104951, 104960, 112475, 134441–42, 138218–19, 138855; MBM 1929, 1931; MSB 31464, 44451–52, 58361; MVZ 9814, 26661, 41139, 41700, 52012, 52014, 72420, 140884, 161441, 176825, 179937, 193437, 204994–95, 205573, 229800, 229958; SDSNH 534–35, 813, 910, 978, 1067, 2606, 2625, 2778, 2814, 2929–31, 2967–69, 3128–30, 3204, 3297–99, 4304, 4652, 4712, 4732, 4831, 4876, 4930, 4935–36, 5073, 5161, 5570, 7095, 7582–83, 8056, 8557, 8559, 8681, 8703, 8801, 9523, 10023, 10338–39, 11917, 12083, 13366, 16724, 19713–14, 20196, 20596, 20737, 20991, 21093, 22414–15, 23066, 23069, 23149, 23151–54, 23225, 23394, 23395, 24003, 24103, 24667, 26045, 26090–91, 27537, 27648, 28570, 28734, 29612, 29654, 31776–78, 31835, 31951, 32774, 33777, 34031, 34956, 35005, 35103, 35466, 36336, 36426, 37463–64, 38995, 39024, 39251, 39815, 39827, 42355, 42970, 44004–09, 44133, 44264, 44359, 44397, 45039, 49677, 49678–80, 59424, 62291, 63918, 67536, 68862; UABC 1033, 667, 746; UAZ 23296, 27600, 35815–16, 39828, 42974, 42976, 43579, 43580, 43945, 44315, 44801, 45888, 54653–54, 55804; UCM 51221; USNM 16353, 115689, 161166, 205523–24, 222787, 225372–73, 239261, 246618, 248137, 253067–68, 307996, 335504; UTA 50748, 51392, 51431, 51444, 53198; UTEP 11450, 12147. MZFC uncatalogued (2 specimens); UTA uncatalogued (3 specimens).

*Crotalus stephensi* (50): BYU 5177–78; CAS 192752, 192757; LACM 36696, 63974, 104931, 134440, 134443; MBM 1959, 3131, 3154, 3160; MVZ 6699, 19344, 228701, 228703; SDSNH 2114, 2970, 4942, 8512, 20986, 21130, 22250, 32519, 32668, 32751, 35117–18, 35122, 35127, 35129, 35131–32, 35141, 35146–47, 35156; TNHC 15329; UAZ 27599; USNM 18662, 18665, 18671–72, 198137, 307994; UTA 51441–42, 53201.

*Crotalus tigris* (84): ASU 5037, 6690, 6737, 22073, 22272, 22861, 23208, 33150, 33163, 33192, 33224, 33309, 33320; CAS 92265, 103472, 192769; IBH 1724; KU 155525; LACM 25178, 105106–07, 105109–10, 144360; MSB 56030; MVZ 54622, 74699, 79234; SDSNH 787, 3132, 3237–38, 3240–43, 17940, 34457, 49715, 49916–17; TNHC 30991, 34903; UAZ 27801, 27804, 27807, 27815, 27818–20, 27822, 27824, 27826–27, 27845–48, 33126, 35820, 36516, 36519, 38206, 39771, 40084, 41947, 45909, 52169, 53554, 53657, 56930; USNM 80070, 156808, 160399, 222085–86, 238291, 246635; UTA 32248, 54073; UTEP 12324, 18765. UTA uncatalogued (1 specimen).
